# Case Report: Serial stereotactic radiotherapy in lieu of systemic therapy change for oligoprogressive breast cancer: eight courses to 17 metastases

**DOI:** 10.3389/fonc.2026.1641876

**Published:** 2026-03-25

**Authors:** Saad Ashraf, Michelle White, Neda Haghighi, Shankar Siva, Keelan Byrne, Michael Liu, Steven David

**Affiliations:** 1 Monash Health, Melbourne, VIC, Australia; 2 Department of Medical Oncology, Monash Cancer Centre, Melbourne, VIC, Australia; 3 Faculty of Medicine, Nursing and Health Sciences, Monash University, Melbourne, VIC, Australia; 4 Department of Radiation Oncology, Peter MacCallum Cancer Centre, Melbourne, VIC, Australia; 5 Sir Peter MacCallum Department of Oncology, The University of Melbourne, Melbourne, VIC, Australia

**Keywords:** breast cancer, metastatic, oligoprogressive, radiotherapy, stereotactic ablative body radiotherapy (SABR), stereotactic body radiation therapy (SBRT), stereotactic radiosurgery (SRS)

## Abstract

Stereotactic ablative body radiotherapy (SABR) is emerging as a strategy for treating oligoprogressive disease in lieu of changing systemic therapy. We report the case of a patient with oligoprogressive breast cancer who received seven sequential rounds of SABR and one course of stereotactic radiosurgery to 17 metastases over 5 years, delaying a change in systemic therapy on eight occasions. The patient’s age, performance status, and treatment preferences guided this unique treatment journey, emphasising quality of life. In limited trials, SABR has not demonstrated improvement in overall survival. Nevertheless, early evidence, including this case of oligoprogression, supports its role in delaying systemic therapy.

## Introduction

Oligometastatic disease refers to an intermediate stage of cancer spread, typically defined by one to five metastases. The key distinction from widespread metastases lies in the potential for cure with targeted therapy, contrasted with the historical view of metastatic disease as systemic and incurable. “Oligoprogressive” disease is a subset in which one or a few metastases progress, potentially representing resistant subclones, while other sites remain controlled on systemic therapy (1).

Stereotactic ablative body radiotherapy (SABR) is an established therapeutic strategy for oligometastatic disease, providing local control and potentially curative treatment. SABR delivers highly focused radiation, often in one or two sessions, with minimal toxicity (2). Compared with other metastasis-directed therapies, such as surgery, SABR can target a wide range of anatomical sites with low morbidity.

In metastatic breast cancer, systemic therapy tailored to tumour biology remains the mainstay of treatment, with therapy typically altered upon disease progression (3). Historically, the role of radiotherapy in metastatic disease has been limited to palliative treatments for symptom control. SABR allows elimination of oligoprogressive metastases in patients whose disease is otherwise responding, thereby delaying the need to change systemic therapy. This approach has shown mixed results, potentially due to patient demographics, trial design, and disease histology, highlighting the importance of careful patient selection, which will be discussed in greater detail in the Discussion.

**Figure 1:**
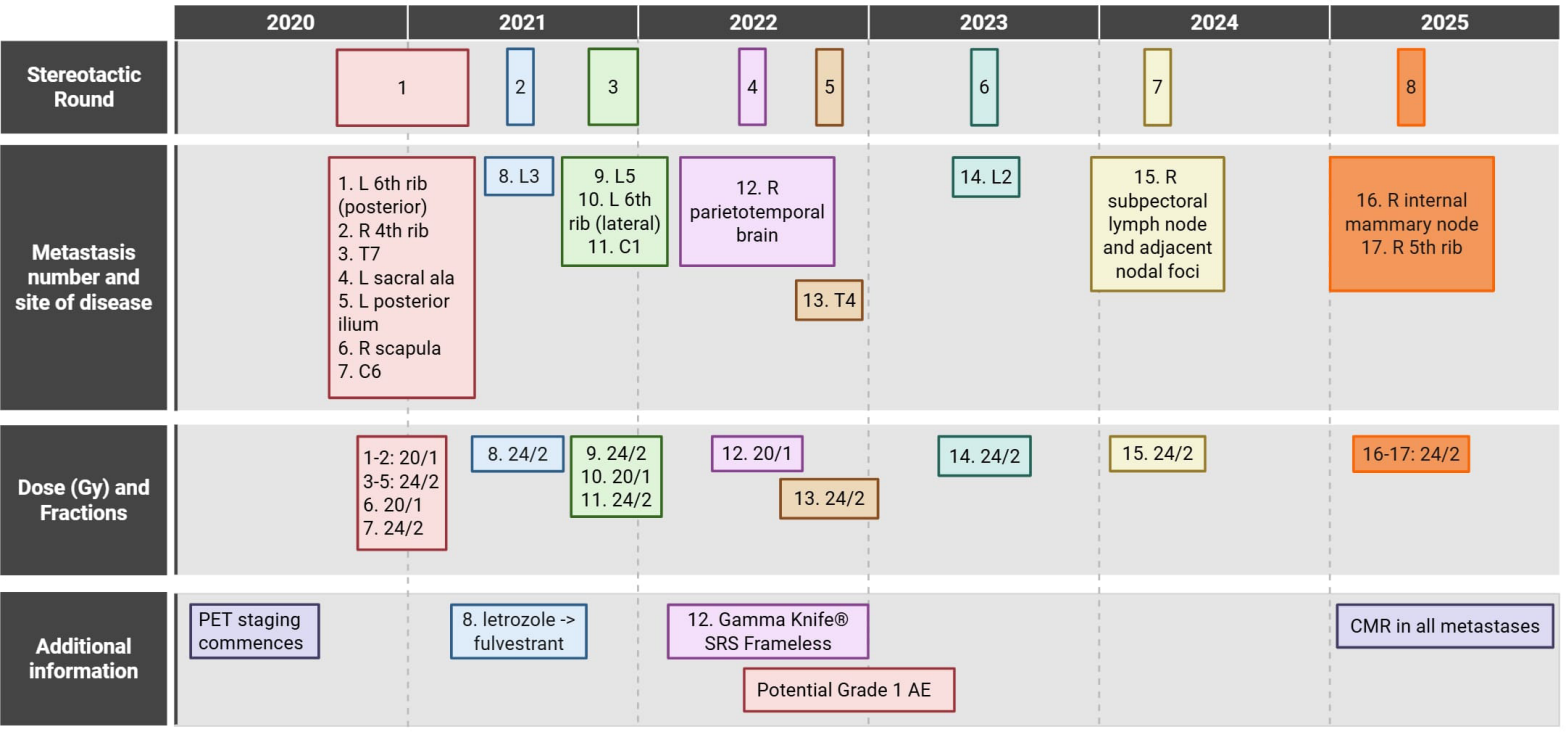
Timeline of stereotactic radiotherapy treatments by stereotactic round, metastasis number, site of disease, and dose/fraction. “Stereotactic rounds” are grouped according to the PET staging scan that detected the relevant metastases. “Metastasis number” is defined as starting from the first stereotactic treatment (i.e., excluding initial VMAT in 2017). All stereotactic treatments were reviewed and discussed at the Peter MacCallum multisite chart round and were treated according to institutional protocols.

**Figure 2:**
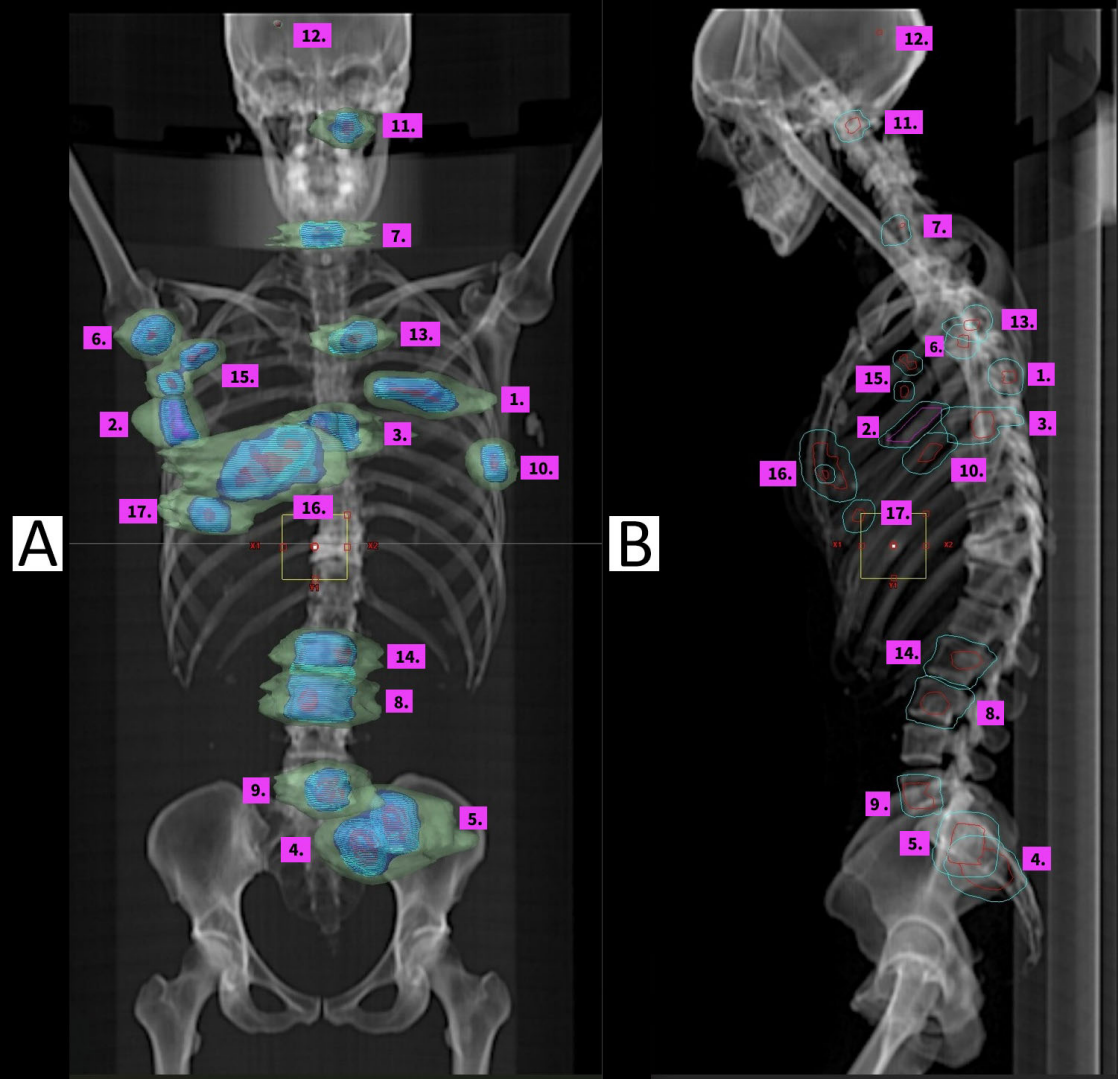
Coronal **(A)** and sagittal **(B)** scout-view reconstructions of the skeleton with overlaid dose lines from each stereotactic treatment. Metastases are numbered in order of treatment; see **Figure 1** for the timeline. **(A)** Light blue indicates the prescribed dose at the 100% isodose level, with light green at the 50% isodose level. **(B)** The central red line represents the gross target volume (GTV), and the outer cyan line represents the planning target volume (PTV).

We report a patient in whom the “serial stereotactic” approach was employed to delay a change in therapy on eight occasions over 5 years for 17 metastatic deposits, while maintaining an excellent quality of life (QOL). To our knowledge, this is the first case in which this number of oligoprogressive events has been managed with metastasis-directed therapy. **Figure 1** shows the treatment timeline, including stereotactic round, location, and dose/fraction of each metastasis, with **Figure 2** visualising each metastatic deposit in a single overlay image.

## Case presentation

A 35-year-old female patient was diagnosed in 2017 with *de novo* metastatic breast cancer following investigation for unresolved back pain. Core biopsy demonstrated an oestrogen receptor-positive (ER^+^), progesterone receptor-positive (PR^+^), and human epidermal growth factor receptor 2-amplified (HER2^+^) carcinoma, with an Allred score of 7. Given the presence of metastatic disease, the patient was not formally staged. Initial metastatic spread involved the right seventh rib, C2–3, and T10–12, the latter being the only symptomatic sites. She received systemic therapy with eight cycles of paclitaxel, along with trastuzumab, pertuzumab, and denosumab. Additionally, palliative radiotherapy via volumetric modulated arc therapy (VMAT) was delivered to T10–12 with good effect. Upon completion of paclitaxel, she continued trastuzumab and pertuzumab and commenced goserelin and letrozole. Following a period of progression-free follow-up, she relocated to another city, where she has since received her remaining treatments.

In 2019, she developed disease progression at the primary site. Given her identified CHEK2 mutation, she elected to undergo a double mastectomy, which revealed multifocal invasive carcinoma in the right breast, the largest lesion measuring 18 mm, which was ER^+^, PR^−^, and HER2^+^, with an Allred score of 7. A sentinel lymph node biopsy demonstrated isolated tumour cells in one of four lymph nodes, and the left breast was free of *in situ* or invasive disease.

In late 2020, staging switched from computed tomography and bone scan to positron emission tomography (PET), which demonstrated seven metabolically active sites. A multidisciplinary meeting recommended SABR to the only symptomatic metastasis in the left sixth rib, which showed a good treatment response on 6-week follow-up PET. Over 12 months, a further 10 metastases were treated with SABR across three rounds of treatment, each demonstrating complete metabolic response (CMR) according to Positron Emission Tomography Response Criteria in Solid Tumors (PERCIST) criteria, which supported delaying systemic therapy progression. However, due to L3 oligoprogression in “round 2”, the patient’s endocrine therapy was switched from letrozole to fulvestrant. Given the absence of a reliable soft tissue component in the bony metastases, response was assessed on PERCIST 1.0 rather than standard Response Evaluation Criteria In Solid Tumors (RECIST) 1.1 (4, 5).

**Figure 3:**
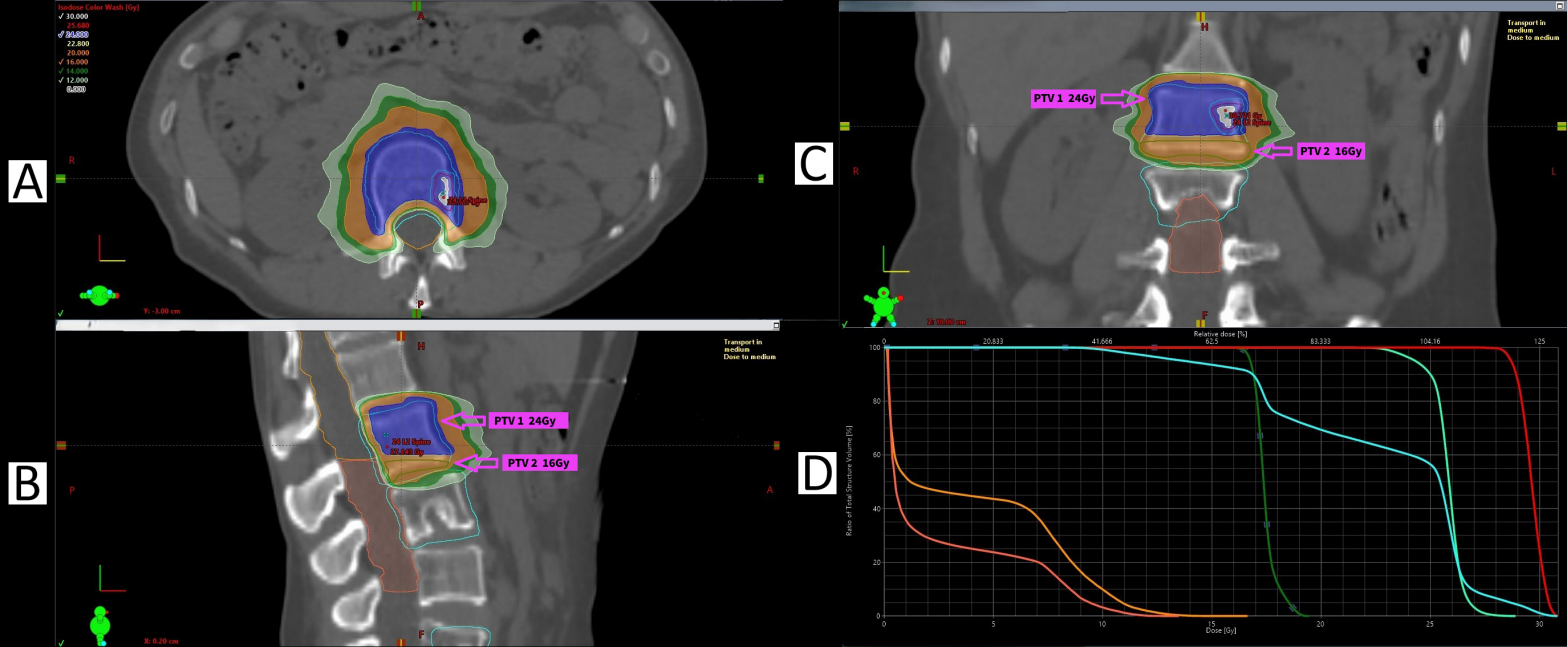
SABR planning for the L2 metastasis (number 14, treated in round 6). **(A)** Axial image demonstrating 24 Gy (shaded blue) coverage of the GTV and PTV, with 14 Gy (shaded orange) sparing the spinal canal at the level of the GTV. **(B)** Sagittal image demonstrating spinal canal dose split into superior section (orange outline), limited to 14 Gy (shaded orange), and inferior section (shaded pink), limited to 12 Gy (shaded light green). **(C)** Coronal image demonstrating PTV 24 Gy splits into two sub-PTVs: PTV1 covered by 24 Gy (shaded blue) and PTV2 covered by 16 Gy (shaded orange). PTV2 was adjusted due to prior irradiation from the L3 metastasis (number 8, treated in June 2021. **(D)** Dose-volume histogram demonstrating L2 vertebral body PTV (cyan) split into two dose levels: 24 Gy (light green) and 16 Gy (dark green). Spinal canal superior (orange) limited to 14 Gy, spinal canal inferior (pink) limited to 12 Gy.

In 2022, a solitary 2-mm right parietotemporal metastasis was identified on magnetic resonance imaging (MRI), which progressed to 4 mm on short-term follow-up, confirming the diagnosis of brain metastasis and representing the first nonbone metastatic site. It was treated with Gamma Knife^®^ stereotactic radiosurgery (SRS) Frameless to 20 Gy in one fraction. On 3-month and subsequent follow-up MRIs, the treated metastasis was not visualised, with no other new brain metastases noted, demonstrating a complete response (CR) per RECIST 1.1—the fourth instance in which the serial stereotactic approach justified delaying systemic treatment progression due to disease control in other sites. Two further treatments delayed treatment progression a fifth and sixth time, with treatment planning for the L2 metastasis shown in **Figure 3**.

In 2024, oligoprogressive disease was noted in the right subpectoral lymph node and adjacent likely nodal foci. Following a multidisciplinary meeting and extensive patient discussion, it was decided to proceed with SABR to avoid the potential toxicity of the planned next line of systemic therapy, trastuzumab deruxtecan (TDxD). Acknowledging risks to nearby organs at risk, including the brachial plexus, she was treated with 24 Gy in two fractions. Similarly, in 2025, upon noting oligoprogression in the right internal mammary node and right fifth rib, SABR was chosen over TDxD—the eighth instance in which this approach justified delaying systemic therapy progression. On the most recent follow-up imaging, these two metastases again demonstrated CMR. Each metastasis showed CMR or CR on routine follow-up imaging, and there has been no recurrence at any previously treated site.

Throughout her disease course, the patient was actively involved in shared decision-making. With guidance from her clinical team, she carefully weighed the potential toxicities of altering her well-tolerated systemic therapy against the option of treating oligoprogressive metastases with local ablative therapy while continuing the same regimen. She reported that she found this strategy, and her involvement in it, both effective and reassuring.

The patient maintained an excellent QOL, remaining Eastern Cooperative Oncology Group (ECOG) performance status 0 throughout, raising her young children, and exercising regularly. All treatments were completed in the Australian public hospital setting, and the patient did not pay for any radiotherapy treatments out of pocket. In November 2022, she developed early right frozen shoulder-like symptoms, which were treated with steroid injection as well as regular physiotherapy. This may be attributable to the right scapular SABR treatment in February 2021, as the 50% dose fall-off slightly overlapped with a small portion of the glenohumeral joint, representing a possible grade 1 adverse effect per the Common Terminology Criteria for Adverse Events (CTCAE) Version 5.0 (6). To date, she has not reported any long-term side effects attributable to radiotherapy. She also did not experience significant pain, occasionally requiring paracetamol and nonsteroidal anti-inflammatory medication, but not opiate agents, thereby managing with “Step 1” of the World Health Organisation Analgesia Ladder (7). Overall, the majority of the patient’s symptoms and possible adverse effects were transient and resolved spontaneously. During a recent review, she was noted to be “fit, well, and completely asymptomatic from any sites of disease currently”.

Dose and fractionation selection was based on institutional protocols and the author’s experience in clinical trials for oligoprogression, with each metastasis treated using the highest dose achievable within organ-at-risk tolerances (2, 8). Current institutional policy for creating Clinical Target Volume (CTV) for bony metastases is to add 5 mm to the gross target volume (GTV) and crop within anatomical boundaries, with a further 5 mm added to create the planning target volume (PTV). This approach was implemented because institutional recurrence has been observed at bony margins on retrospective review, and a CTV expansion was postulated to help address subclinical disease (9). Each treatment was reviewed at the Peter MacCallum Cancer Centre’s SABR chart round, with local dose constraints broadly based on guidelines published by Timmerman, with subsequent modifications (10). The decision to use 20 Gy in one fraction was applied for smaller metastases with no overlap, such as the right scapula or solitary brain metastasis. In later treatments, 24 Gy in two fractions was utilised, primarily due to overlap with previous treatments; for example, the R subpectoral lymph node (metastasis 15) was in a region of multiple prior treatments, as seen in **Figure 2**, and fractionation was preferred based on institutional trial-based safety data (11). Another example is the L2/L3 overlap in treated metastases eight and 14, where L3 was treated with fractionation due to the overlap. The presence of larger metastases also supported using 24 Gy in two fractions, as this results in a lower biologically effective dose (BED), making treatment theoretically safer and better tolerated. An overlay image demonstrating all sites of irradiation is shown in **Figure 2**. All treatments were conducted on a Varian™ TrueBeam™ linear accelerator utilising VMAT technology with photon beam energies of 6 and 10 megavolts.

**Figure 4:**
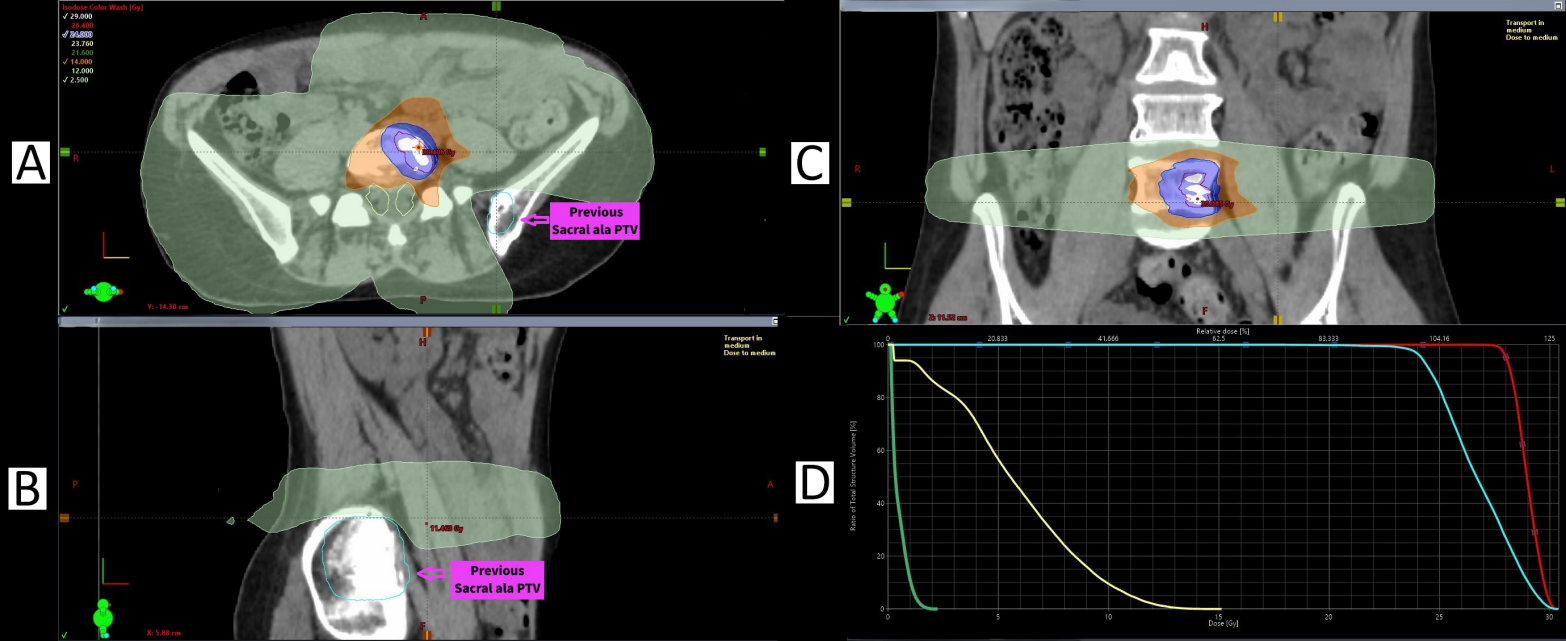
SABR planning for the L5 metastasis (number 9, treated in round 3). **(A)** Axial image demonstrating 24 Gy (shaded blue) coverage of the L5 PTV, with 14 Gy (shaded orange) limiting dose to the cauda equina (yellow outline). The previously treated left sacral ala metastasis (cyan outline, metastasis number 4, treated in round 1) is spared, as demonstrated by avoidance of the 2.5-Gy isodose level (shaded light green). **(B)** Sagittal image showing the 2.5-Gy dose level completely avoiding the previous SABR treatment to the left sacral ala metastasis. **(C)** Coronal image showing full superior and inferior coverage of the PTV of the L5 vertebral body. **(D)** Dose-volume histogram showing full coverage of the L5 PTV (cyan) at 24 Gy, previous sacral ala PTV (light green) limited to a maximum dose of 2.5 Gy, and the cauda equina limited to 14 Gy.

Specific modifications to treatment planning are shown in **Figures 3**, **4**. **Figure 3** illustrates how planning for the L2 metastasis treated in round six was adjusted due to prior irradiation of the L3 metastasis in round two. Two PTVs were used, with the inferior portion of the L2 vertebral body covered by a 16-Gy PTV instead of 24 Gy elsewhere, while 100% of the GTV received 24 Gy. The maximum BED is the small overlap between both treatments, using an α/β of 3, was 178.67 Gy. The spinal cord dose was divided into a superior portion, limited to 14 Gy (due to absence of prior irradiation), and an inferior portion, limited to 12 Gy, to minimise potential toxicity. **Figure 4** examines planning for the L5 metastasis treated in round three, showing how SABR’s highly conformal nature avoids irradiating the previously left sacral ala metastasis from round one, with the latter dose limited to a maximum of 2.5 Gy and the cauda equina to 14 Gy. These examples illustrate how SABR can simultaneously achieve adequate GTV and PTV coverage while sparing previously irradiated regions and adhering to organ-at-risk dose limitations.

## Discussion

Several trials have investigated the use of SABR in oligoprogressive disease, showing varied results across different cancer types and patient populations. The Consolidative Use of Radiotherapy to Block (CURB) randomised phase II trial evaluated SABR in patients with breast cancer and non-small cell lung cancer (NSCLC), demonstrating improvement in progression-free survival (PFS) in the NSCLC cohort (2.2 months vs. 10 months) but not in the breast cohort (4.2 months vs. 4.4 months) (12). The breast cohort included a large proportion of patients pretreated with chemotherapy and patients with TNBC, both factors associated with lower survival. The STOP phase II trial randomised 90 patients with oligoprogressive solid tumours to SOC with or without SABR, showing improvements in local control but no significant differences in PFS or overall survival (OS) (13). However, the findings were limited by a high rate of crossover, with 71% of the control group receiving radiotherapy after randomisation, of which 19% involved high-dose or ablative therapy. To mitigate slow accrual, systemic therapy decisions were left to the physician’s discretion at randomisation. The heterogeneous patient population and treatment pathways may have obscured the benefits from SABR that could be more apparent with stricter patient selection. The results of the HALT randomised phase II/III trial will further clarify the efficacy of SABR in oligoprogressive disease, focusing on mutation-positive NSCLC patients receiving tyrosine-kinase inhibitors (14). Notably, these trials used progression-free survival as the primary endpoint, which may underestimate the value of SABR in delaying changes in systemic therapy. As demonstrated in this case, multiple courses of SABR can remain effective beyond initial progression, providing continued benefit without requiring changes to systemic treatment.

The AVATAR single-arm phase II trial evaluated the impact of SABR in delaying changes to systemic therapy in oligoprogressive ER^+^/HER2^−^ breast cancer, reporting a median PFS of 5.2 months and a median modified PFS (mPFS) of 9.9 months. In this trial, 44% and 36% of patients continued their current therapy for 12 and 24 months, respectively (15). The study specifically analysed an additional endpoint, mPFS, defined as progression not amenable to further SABR treatments—a practical metric for clinicians and patients when discussing treatment options and expected prognosis. Notably, selected patients in AVATAR received SABR for two or three rounds instead of changing systemic therapy. The RADIANT single-arm phase II study evaluated SABR in metastatic genitourinary, breast, and gastrointestinal cancers (16). Across all cancer types, a change in systemic therapy within 1 year occurred in 47% of patients (41% in breast), with PFS observed in 32% and a median PFS of 4.7 months (6.5 months in breast). The higher PFS in AVATAR and RADIANT compared with CURB may be attributable to their HR^+^, HER2^−^ breast cancer population, with AVATAR further distinguished by stricter criteria regarding systemic therapy; these factors also limit their external validity. Overall, despite differences in trial design and patient demographics, evidence from oligoprogressive trials suggests that SABR can improve PFS, with benefits appearing to correlate with patient characteristics.

Although PFS and OS are commonly used outcomes in metastatic cancer research, they may not fully capture a patient’s goals or experience of well-being. Regarding OS, it is important to distinguish between maintaining reasonable QOL and functional status vs. merely prolonging life, as OS gains can be offset by significant treatment-related morbidity. Furthermore, reliance on PFS in clinical decision-making may lead to premature changes in systemic therapy. In this case, the decision to pursue serial stereotactic therapy was strongly influenced by the patient’s sustained performance status (ECOG 0 throughout) and her strong preference to defer more toxic systemic therapies for as long as possible, thereby preserving her QOL.

Modern insights into tumour biology support a “resistant subclone theory”, whereby metastatic tumours consist of multiple cell lines, some of which possess inherent or acquired resistance to systemic therapy (17). Oligoprogressive sites are thought to represent resistant cell lines that pose the greatest risk of progressing to widespread metastatic disease. Martínez-Jiménez et al. found that late-stage metastases differed significantly from initial primary cell lines in prostate, breast, thyroid, renal clear cell, and pancreatic neuroendocrine carcinomas (18). Multiple resistance genes were identified as occurring specifically in the context of treatment resistance, including ESR1 in breast cancer treated with aromatase inhibitors. Yates et al. sequenced 17 metastatic breast cancer patients at various time points, similarly finding ESR1 mutations in late metastasis, as well as other “driver” mutations in the SWI/SNF or in JAK–STAT signalling, which appear to occur specifically following endocrine therapy (19). By early identification and ablation of these clones, it is theorised that SABR delays the time until uncontrollable resistant metastatic spread. Although biopsies on oligoprogressive sites were not undertaken in this patient, the indolent manner of progression, combined with the excellent response to hormonal therapy, lends support to the resistant subclone hypothesis and the efficacy of SABR in delaying the time to change in systemic therapy.

The decision to consider SABR beyond two or three oligoprogression stages is guided by several factors, such as patient age and ECOG status, disease tempo, safety considerations, and the availability of healthcare resources such as a multidisciplinary cancer service and imaging services. Among breast cancer subtypes, ER^+^ and HER2^+^ breast cancers often respond well to multiple lines of systemic therapy for prolonged periods, making these malignancies well-suited to the approach reported in this case. Furthermore, though dependent on anatomical site, serial rounds of SABR result in an increased volume of irradiated tissue, which may impact the feasibility of further treatments because of dose constraints, particularly to organs at risk. **Figure 4** illustrates how SABR’s high degree of dose conformity permitted essentially complete avoidance of a previously treated metastasis. **Figure 3** further demonstrates that despite dose modifications being necessary due to a closely located previously treated metastasis, SABR treatment remained feasible while limiting the dose to organs at risk, particularly the spinal cord.

Long-term toxicities of SABR include fractures, lung fibrosis, rectal irritation/bleeding, and bone marrow damage, with the latter potentially limiting certain chemotherapies (20–22). Although this was a risk in this patient, given the large number of bony sites treated, marrow suppression did not occur. Two possible grade 1 AEs occurred in this patient; however, these were transient. Notably, the patient is currently asymptomatic from any site of disease, attributable to the low morbidity associated with stereotactic treatment.

The likelihood of selection bias and diminishing returns should strongly guide the decision to alter treatment, with systemic therapy remaining the backbone of metastatic breast cancer management. In this patient, metastases were oligoprogressive, demonstrated CMR on follow-up imaging, and no symptomatic visceral metastases were noted; each of these factors justified not switching systemic therapy despite progressive disease. However, selection bias warrants consideration, as optimal candidates for serial stereotactic treatment—more indolent tumours and fewer oligometastases—may have more favourable tumour biology and response to systemic therapy, conferring better outcomes irrespective of radiotherapy use. As an isolated case, this report intends to demonstrate the feasibility of the serial stereotactic approach; however, there is a need for randomised trials and multivariate analyses to investigate selection and other biases and to demonstrate that this approach is oncologically safe and beneficial.

The serial stereotactic approach may increase healthcare resource burden due to frequent radiotherapy consultations, planning, treatments, and follow-up imaging. However, Qu et al. demonstrated SABR is cost-effective in oligometastatic cancer when considering QOL, with Lourenco et al. demonstrating that drug therapies were more than 10 times the price of SABR (23, 24). Furthermore, this approach is appropriate only for select patients and is therefore unlikely to change existing workflows significantly. Additionally, chemotherapy is substantially more resource burdensome because of expensive medications, frequent monitoring, and the management of side effects (25).

## Conclusion

To our knowledge, this is one of, if not the first, reported cases using a serial stereotactic approach. In this case, the patients received seven SABR treatments to 16 metastases and one SRS to a brain metastasis, with a goal of delaying a change in systemic therapy. This approach shows promise as a combination therapy alongside systemic therapy in oligoprogressive disease. Although trials have shown mixed results, outcomes appear to depend on tumour type, biology, trial design, and other patient demographics, with this patient’s HER2-amplified status potentially contributing to her excellent treatment response. In appropriately selected patients, this approach appears to be a feasible and cost-effective option; however, further research is needed to validate findings in larger, prospective, and randomised trials.

## Data Availability

The original contributions presented in the study are included in the article/supplementary material. Further inquiries can be directed to the corresponding author.
